# Pregnancy in Patients with Non-cirrhotic Portal Hypertension: A Literature Review

**DOI:** 10.1055/s-0042-1748973

**Published:** 2022-06-27

**Authors:** Suprabhat Giri, Shradhanjali Sahoo

**Affiliations:** 1 Department of Gastroenterology, Seth GS Medical College and KEM Hospital, Mumbai, Maharashtra, India; 2 Department of Maternal and Fetal Medicine, Fernandez Hospital, Hyderabad, Telangana, India

**Keywords:** portal hypertension, esophageal and gastric varices, pregnancy outcomes, EHPVO, NCPF, hipertensão portal, varizes esofágicas e gástricas, resultados da gravidez, EHPVO, NCPF

## Abstract

Pregnancy in non-cirrhotic portal hypertension (NCPH) is an uncommon condition. Its management is challenging both to the obstetricians as well as to the gastroenterologists due to the lack of more extensive studies and standard clinical practice guidelines. These patients are at increased risk of portal hypertension (PTH) complications, especially variceal bleeding, and with an increased incidence of adverse maternal and fetal outcomes. Hence, a multidisciplinary approach is required for management of pregnancy in NCPH. This short review describes the different aspects of pregnancy with NCPH, emphasizing specific strategies for preventing and managing PTH from the preconceptional period to postpartum.

## Resumo

A gravidez na hipertensão portal não cirrótica (HPNC) é uma condição incomum. Seu manejo é desafiador tanto para os obstetras quanto para os gastroenterologistas devido à falta de estudos mais extensos e diretrizes de prática clínica padrão. Esses pacientes apresentam risco aumentado de complicações da hipertensão portal (PTH) especialmente sangramento por varizes e têm maior incidência de desfechos maternos e fetais adversos. Portanto uma abordagem multidisciplinar é necessária para o manejo da gravidez na NCPH. Esta breve revisão descreve os diferentes aspectos da gravidez com HPNC enfatizando estratégias específicas para prevenção e manejo do PTH desde o período pré-concepcional até o pós-parto.

## Introduction

Portal hypertension (PHT) is defined as a pressure gradient of 3.5 mm Hg between the portal vein (PV) and inferior vena cava (IVC), which can be due to cirrhosis or have a non-cirrhotic etiology. Non-cirrhotic portal hypertension (NCPH) is common in developing countries, 1 among which non-cirrhotic portal fibrosis (NCPF) and extrahepatic PV obstruction (EHPVO) are the two most common causes, 1 with other less common etiologies as enumerated in Chart 1 . 2 Compared with patients with cirrhosis, NCPH is not associated with significant hepatic dysfunction. Hence, fertility in women with NCPH is usually normal compared with patients with cirrhosis. Association of NCPH in pregnancy is an infrequent scenario, but when it occurs, it can lead to a complex clinical situation due to associated PHT and risk of variceal bleed. There is a paucity of data with regard to the prevalence of NCPH in pregnant patients, and the majority of data reported are from India 3 4 5 6 7 8 9 10 with few multicentric studies from Europe. 11 12 As of yet, there are no definitive guidelines regarding the management of pregnancy in NCPH. Hence, this narrative review aims at summarizing the current data on pregnancy outcomes in patients with NCPH and the management of portal hypertension and its complications during pregnancy.

**Chart 1:** Etiologies of non-cirrhotic portal hypertension (NCPH)

Pre-sinusoidal	Sinusoidal	Post-sinusoidal
o Portal vein obstruction o Porto-sinusoidal vascular disease (PSVD) o Schistosomiasis o Arteriovenous fistulas o Polycystic liver disease o Congenital hepatic fibrosis o Biliary diseases (primary biliary cirrhosis; primary sclerosing cholangitis)	o Drug-induced o Acute fatty liver of pregnancy o Alcoholic liver damage o Non-alcoholic steatohepatitis o Viral hepatitis o Amyloidosis o Infiltrative diseases o Gaucher's disease o Visceral leishmaniasis	o Budd-Chiari syndrome o Veno-occlusive disease o Primary vascular malignancies o Hypervitaminosis A o Epithelioid hemangioendothelioma and angiosarcoma

**Source:** Gioia et al. (2020). 2

## Methods

A literature search was conducted on PubMed using the dates of 1990 to 2021 with the following medical subject headings (MeSH) terms: *Hypertension* , *Portal* [Mesh] AND *Pregnancy* [Mesh]. There was no restriction regarding language as long as study outcomes are mentioned in the text. The search yielded a total of 249 results which were independently reviewed by two authors, and it was decided by consensus which articles to incorporate in this review. We also searched the bibliography of the included studies for any relevant studies. The studies describing outcomes in patients with cirrhosis were excluded.

## Hemodynamic Changes during Pregnancy

**Fig. 1:**
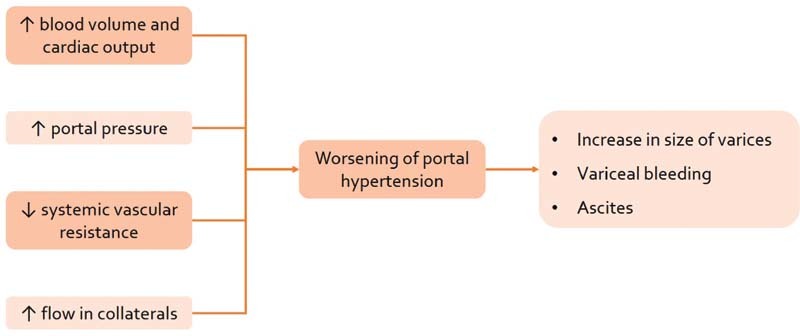
Effect of pregnancy hemodynamics on portal hypertension. **Source:** López-Méndez and Avila-Escobedo (2006). 13

Pregnancy is associated with several changes in hemodynamic and physiological parameters due to the growing needs of the fetus. An increase in plasma volume by 40 to 50% is one of the first changes to occur. Increases in stroke volume and heart rate result in a 30 to 50% increase in maternal cardiac output. The effect of progesterone and placental bed development contributes to the reduced systemic vascular resistance. 13 Consequently, these hemodynamic alterations lead to hyperdynamic circulation ( Fig. 1 ) 13 with widened pulse pressure, ultimately leading to the worsening of PTH and increased risk of variceal bleeding. So, the primary aim of the management of NCPH in pregnancy is to reduce PTH to prevent complications and improve maternal and fetal outcomes.

## Pregnancy Outcomes

### Maternal Outcomes

Variceal bleeding remains the most devastating complication of NCPH in pregnancy. Though variceal bleeding may occur at any point during a pregnancy, it is most common during the second and third trimesters and during the second stage of labor. Studies have shown that variceal bleeding occurs in 4.3 to 34% of pregnancies. 3 4 5 6 8 9 10 11 But pregnancies in which the PHT was diagnosed and managed prior to conception have lower variceal bleeding rates. 6 9 Variceal bleeding during pregnancy has been associated with abortion, preterm labor, and maternal death. 9 14 However, a recent study has shown comparable maternal and perinatal outcomes between patients who developed variceal bleeding and those who did not. 5 This might be due to an improvement in endoscopic techniques and wider availability of endoscopic facilities. Pregnant patients with NCPH tolerate variceal bleeding better, with 2 to 6% mortality rates, compared with cirrhotics, in whom the mortality rate ranges from 18 to 50%. 15 Ascites is uncommon in pregnant patients with NCPH compared with cirrhotic pregnant patients. However, ascites can still be seen in patients with non-cirrhotic PHT, with a reported incidence of 0.8 to 10% during pregnancy and usually disappear after delivery. 3 4 9 Reported causes of maternal mortality include postpartum hemorrhage (PPH) and preeclampsia with hemolysis, elevated liver enzymes, low platelet count (HELLP) syndrome and disseminated intravascular coagulation. 5 10 Rupture of splenic artery aneurysm is a rare but life-threatening complication that can occur in pregnancy with PHT caused by high estrogen levels, presence of splenomegaly, increased blood flow from pregnancy, and PHT. 16 The risk of rupture is highest in the third trimester and is associated with a very high maternal and fetal mortality rate of up to 75% and 95%, respectively. 16 17 18 Hence, screening for splenic artery aneurysms is recommended prior to conception with definitive endovascular or surgical management, if present. 18

### Fetal Outcomes

Pregnancy in NCPH is associated with adverse perinatal outcomes with increased rates of spontaneous abortion, premature delivery, small size for gestational age, stillbirth, and perinatal mortality. The reported incidence rate of spontaneous abortion in various studies is up to 23.8% of patients with NCPH. 3 4 5 6 7 8 9 10 11 Development of variceal bleeding during pregnancy in NCPH is well tolerated by the mother but poses a greater risk for the fetus. The incidence of preterm birth has been reported to be 10 to 37.5% in NCPH patients. 4 5 6 7 9 10 11 12 Neonatal mortality is seen in up to 16% of pregnancies. 4 5 6 7 8 9 10 11

## Management of Portal Hypertension in Pregnancy

### Preconceptional Counselling

Detailed preconceptional counselling should be done for all women with NCPH. Patients should be oriented about the effect of pregnancy on PHT, the risk of complications during pregnancy, and the impact of drug therapy on the fetus. Patients should undergo a surveillance endoscopy prior to preconception for planning appropriate management of PTH. Prophylaxis for variceal bleeding can be achieved through either endoscopic variceal ligation (EVL) or β-blockers. Prior history of variceal bleeding is a risk factor for bleeding during subsequent pregnancies. 5 Hence, combination therapy with EVL and β-blocker is preferred for patients with varices and a previous history of variceal bleed.

### Antenatal Management

If initial endoscopy was not performed preconceptionally and the patient presents for follow-up after conception, then endoscopy should be performed to assess the variceal status and plan further management. Despite limited studies on the safety of endoscopy in pregnant patients, it can be performed safely if the risk, benefit, and clinical indications are assessed carefully. As per the ACG guidelines on liver disease and pregnancy, the optimal time to screen for esophageal varices is in the second trimester, right after organogenesis has completed in the first trimester, and before delivery. Propofol is the preferred sedation for endoscopy due to its Food and Drug Administration (FDA) pregnancy category B. Meperidine can also be used but is less preferred due to pregnancy category C. 19 Either EVL or β-blockers can be used for the control of varices. Non-selective β-blockers (propranolol, nadolol) and combined non-selective β-blockers with α1-adrenergic receptor antagonist (carvedilol) are pregnancy category C drugs. The use of β-blocker in pregnancy has been associated with an increased risk of congenital malformations, fetal growth restriction, fetal bradycardia, and neonatal hypoglycemia. 20 21 However, the benefit of using β-blocker outweighs the risk in patients with NCPH and should be continued in patients with large varices or small varices with red color signs (RCS). Patients with NCPH are also at risk for anemia, due to associated hypersplenism, and should be treated actively to reduce adverse outcomes like pre term labor and low birth weight. Hence, two weekly maternal and fetal monitoring with monthly hematological assessment is advised in these patients. 14

### Peripartum Management

Vaginal delivery is preferred, with a shorter second stage of labor, as repeated Valsalva maneuver leads to an increased risk of variceal bleeding. Forceps or vacuum extraction can be considered, if necessary, to shorten the second stage. Prophylactic shortening of the second stage of labor can be done to avoid overstraining by the mother. Multiple studies from India have reported using the vaginal mode of delivery in 50 to 84% of pregnant patients with NCPH. 5 6 7 8 9 The current obstetric pain management relies mainly on neuraxial anesthesia (epidural, spinal, and combined spinal epidural techniques). Among these, epidural analgesia is the preferred option as it can also work if a lower segment caesarean section (LSCS) is required. Neuraxial analgesia can be safely administered if clinically indicated, with a very low risk of spinal epidural hematoma, if the platelet count is 3 70,000/mm ^3^ . 22 There are no recommendations to perform a LSCS for all patients with PHT, and it should be reserved only for obstetric and fetal indications due to a higher risk of postsurgical bleeding in the setting of PHT. There are no studies that have compared the outcome of vaginal delivery versus LSCS in PTH. Thrombocytopenia and anemia are common in patients with PHT and can be severe enough to require preprocedural transfusion. A platelet count of at least 50,000/mm ^3^ is required to perform LSCS safely; hence, a platelet count below this level warrants transfusion before delivery. 23 Severe thrombocytopenia requiring platelet transfusion has been reported in 8.3 to 50% of patients. 6 7 11

### Postpartum Management

Patients with PHT should be placed on strict postpartum monitoring due to an increased risk of PPH due to associated thrombocytopenia. Around 2.5 to 20% of patients with NCPH develop PPH. 4 5 6 7 9 10 11 12 Postpartum hemorrhage management is the same as that of patients without PTH, including red blood cell units and platelet transfusion, oxytocin drip, and uterine artery embolization or surgery in case of failure of the above. There are no contraindications for breastfeeding in NCPH patients.

### Management of Variceal Bleeding in Pregnancy

**Fig. 2:**
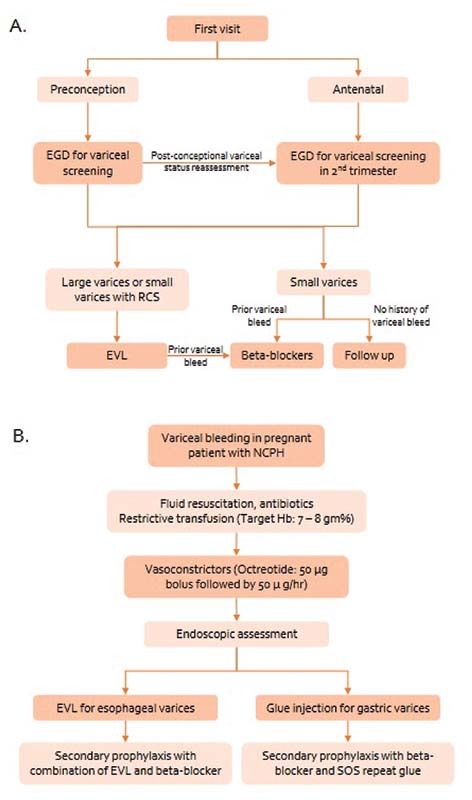
( **A** ) Management of portal hypertension in the preconceptional period and pregnancy, ( **B** ) Management of variceal bleeding in pregnancy. Abbreviations: EGD, esophagogastroduodenoscopy, RCS, red color signs, EVL, endoscopic variceal ligation; NCPH, non-cirrhotic portal hypertension; Hb, hemoglobin.

The management of variceal bleeding in pregnancy is the same as that of other patients, with pharmacological and endoscopic therapy being the mainstay of therapy. Preendoscopic preparation includes immediate resuscitation and hemodynamic stabilization of the pregnant patient. Octreotide (pregnancy category B) is the preferred vasoconstrictor agents in these patients, along with preprocedural antibiotics. 19 Terlipressin is a category D drug, and it also causes increased uterine contractions, leading to reduced uterine blood flow. Hence, the use of terlipressin should be avoided. 19 Endoscopic variceal ligation (EVL) is preferred over endoscopic sclerotherapy due to the lower risk of rebleeding and complications (ulceration, esophageal perforation, and stricture), with a higher rate of variceal eradication. 24 Gastric variceal bleeding can be managed with cyanoacrylate glue injection without any additional risk of complications. 5 Development of variceal bleeding in a patient already on β-blocker warrants combination therapy with EVL and β-blocker for secondary prevention, although there are no direct studies available for pregnant patients. 25 Fig. 2 summarizes the management of PHT and variceal bleeding in pregnancy.

## Conclusion

Compared with patients with cirrhosis, women with NCPH have a better pregnancy outcome. Given the higher incidence of complications associated with PHT, these patients should be managed at a tertiary care level, with a multidisciplinary team including an obstetrician, hepatologist, anesthesiologist, and perinatologist. Variceal bleeding is associated with poor maternal and fetal outcomes; hence, effective control of PHT should be the primary aim of management as patients can still bleed even with NSBB therapy. Future studies are required to evaluate the role of primary prophylaxis with NSBB in patients with small varices as well as the role of combination therapy in secondary prophylaxis.
